# The Effect of Cognitive Impairment on Loneliness in Older Adulthood: Evidence From HRS 2008-2018

**DOI:** 10.1093/geroni/igaa057.1031

**Published:** 2020-12-16

**Authors:** Ji Hyun Lee, Martina Luchetti, Angelina Sutin, Antonio Terracciano

**Affiliations:** Florida State University College of Medicine, Tallahassee, Florida, United States

## Abstract

Background: People experience loneliness when there is a mismatch between desired and actual social interaction. Demographic and health factors have been implicated in loneliness; less is known about the unique association of cognitive impairment on loneliness in older adulthood. Purpose: This study examined the link between cognitive impairment status and level and change in loneliness over a 9-year period and whether it is independent of physical health, depression, and social isolation. We examine the associations for overall and the emotional and social loneliness sub-domains of loneliness. Methods: Data were from the Health and Retirement Study 2008-2018 waves (N = 8,269, age 50+). Cognitive impairment status was categorized using mTICS. Loneliness was measured with 11-item UCLA Loneliness scale. Multilevel modeling was used to analyze the effects of cognitive status on loneliness, controlling for time-varying functional limitation, disease burden, social contact, and depression. Results: Cognitive impairment not dementia (CIND) was associated with higher loneliness (b = .04, p < .001). CIND (b = .03, p = .036) and dementia (b = .09, p = .017) were linked to higher emotional loneliness but were not independent of social isolation and depression. Those with CIND had higher social loneliness (b = .04, p = .016), even after adjusting for covariates. The trajectory of loneliness did not vary by cognitive status. Conclusions: Cognitive impairment is a risk factor for loneliness among older adults. Those with mild cognitive impairment experienced heightened loneliness, especially for social belongingness. Cognitive function should be considered in designing interventions for loneliness.

